# Prevalence of Dental Anomalies Among Orthodontic Patients: A Retrospective Study in Saudi Arabia

**DOI:** 10.7759/cureus.49893

**Published:** 2023-12-04

**Authors:** Felwa S AlHudaithi, Noor A AlDuhayan, Lama N AlJohani, Shouq N AlJohani, Heyam S AlQarni, Mayssa H AlSawadi

**Affiliations:** 1 Preventive Dentistry, Orthodontic Division, College of Medicine and Dentistry, Riyadh Elm University, Riyadh, SAU; 2 Dentistry, College of Medicine and Dentistry, Riyadh Elm University, Riyadh, SAU

**Keywords:** supernumerary tooth, orthodontics, teeth impaction, hyperdontia, hypodontia, dental anomalies

## Abstract

Background: Odontogenic anomalies encompass deviations in dental morphology, orientation, or spatial positioning within the mandibular structures. This study probed the frequency of such dental malformations among orthodontic patients receiving treatment in Riyadh City, Saudi Arabia. Furthermore, the study sought to discern variations in the manifestation of these dental anomalies related to gender and nationality.

Materials and methods: A retrospective analysis was conducted on 384 panoramic radiographs belonging to orthodontic patients (comprising 222 males and 162 females) who sought treatment at orthodontic clinics of a privately owned university hospital in Riyadh City between 2017 and 2019. The patient records were scrutinized for various dental abnormalities, including but not limited to dilacerated teeth, supernumerary teeth, congenital absence of teeth, impactions, hyperdontia, hypodontia, taurodontism, tooth rotation, and amelogenesis imperfecta. The Chi-square test was employed to assess the correlation between the prevalence of dental anomalies and variables such as gender and nationality. A p-value of less than 0.05 was deemed statistically significant for all tests.

Results: Among the assessed sample size of orthodontic patients, dental impactions emerged as the most prevalent dental anomaly, affecting 246 patients (64.1%). This was followed by the occurrence of supernumerary teeth in 31 patients (8.1%), hyperdontia in 29 patients (7.6%), and congenital absence of teeth in 28 patients (7.3%). Other less frequently observed dental irregularities included dilacerated teeth in 23 patients (6%), amelogenesis imperfecta in 12 patients (3.1%), taurodontism in 12 patients (3.1%), and tooth rotations in five patients (1.3%). A statistically significant gender-based disparity was observed, with dental impactions being more prevalent among males (n=154; 69.4%) than females (n=92; 56.8%). Conversely, supernumerary teeth were more prevalent among females (n=24; 14.8%) than males (n=7; 3.2%). No significant variation in the prevalence of dental anomalies was discernible across different nationalities.

Conclusion: Impactions and the presence of supernumerary teeth were the predominant dental anomalies detected among the studied orthodontic patient population. The prevalence of dental anomalies exhibited discernible variations based on gender but not nationality. These disparities could potentially influence orthodontic outcomes, underscoring the necessity for meticulous examination and tailored orthodontic treatment planning.

## Introduction

Odontogenic deviations encompass alterations in the formation, orientation, or spatial positioning of dental structures, mandibular components, or oral tissues. The most frequently occurring odontogenic variations are maxillary canine impaction, hypodontia, and the presence of supernumerary teeth. The presence of non-erupted permanent incisors, dental transposition, macrodontia, microdontia, tooth dilaceration, and ectopic emergence of the first permanent molar are relatively infrequent dental malformations [1,2].

Supernumerary teeth denote the presence of additional dental structures beyond the conventional dentition. In Homo sapiens, the incidence of supernumerary teeth oscillates between 0.1% and 3.8% in permanent dentition and between 0.35% and 0.6% in deciduous dentition, with a higher propensity in males [3]. Hypodontia refers to the congenital absence of one or more teeth, excluding the third molars. Potential complications of this condition may encompass microdontia, prolonged retention of deciduous teeth, delayed eruption of permanent teeth, and limited alveolar development [4].

Maxillary canine impaction refers to the inability of the canine tooth to emerge into its functional position due to obstructions from neighboring bone, dental, or fibrous tissue. A prevalence of 1.7% has been documented for canine impaction in the general population, with a higher incidence observed in patients undergoing orthodontic treatment [5].

Hypodontia, or tooth agenesis, corresponds to the absence of one or more teeth during the developmental stage. The condition can manifest through signs such as microdontia, retained primary teeth, delayed eruption of permanent teeth, and inadequate alveolar development. The frequency of hypodontia varies among different geographical regions and ethnic groups; it is highest in Africa (13.4%), followed by Asia (7%), Europe (7%), and Australia (6.3%), and demonstrates a higher prevalence among females [4, 6]. The teeth most commonly affected by hypodontia, excluding the third molars, are the mandibular second premolars, maxillary lateral incisors, and maxillary second premolars [7].

In a comprehensive dental investigation conducted in the Eastern Province of Saudi Arabia, orthopantomograms (OPGs) were analyzed from a cohort of 1104 individuals, exhibiting an average age of 35.32 with a standard deviation of ±16.63. The study identified developmental anomalies in 401 out of 1104 instances, representing 36.3% of the total study population. The gender distribution of these anomalies appeared skewed, with 33.2% pertaining to male patients and 66.8% to female patients. The anomalies encompassed a range of dental malformations, including dilacerated teeth (30.2%), congenitally missing teeth (24.7%), supernumerary teeth (1.8%), and singular instances of talon cusp and taurodontism (each 0.1%). Of these, pediatric patients represented 1.5% [8].

In a separate study involving 1252 juvenile subjects, a prevalence of distinct dental aberrations was noted in 318 cases (25.39%). The gender distribution comprised 143 females (23.28%) and 175 males (27.42%). Among the number of anomalies, hyperdontia (3.5%) and hypodontia (9.7%) were the most frequently observed in Saudi juveniles. Size-related anomalies included both macrodontia (1.8%) and microdontia (2.6%), while shape deviations encompassed talon cusp, taurodontism, and fusion (1.4%, 1.4%, and 0.8%, respectively). The positional anomalies comprised ectopic eruptions (2.3%) and rotations (0.4%). In terms of structural aberrations, amelogenesis imperfecta (0.3%) and dentinogenesis imperfecta (0.1%) were observed. The clinical indicators of these anomalies may include cyst formation, median diastema, delayed or premature eruption of neighboring teeth, misalignment, crowding, and root resorption of adjacent teeth. Morphological classifications for supernumerary teeth involve conical, tuberculate, supplemental, and odontomas, while location-based categories include mesiodens, paramolar, distomolar, and parapremolar [9].

The presence of dental anomalies can induce perturbations in maxillary and mandibular dental arch lengths and occlusions, thereby complicating orthodontic treatment plans. Hence, establishing the prevalence of these dental anomalies in orthodontic patients is essential for the formulation of effective treatment strategies by orthodontists. To this end, this retrospective radiographic investigation was undertaken with the objective of assessing the prevalence of dental malformations in orthodontic patients from private university-associated hospitals in Riyadh City, Saudi Arabia. We also aimed to investigate potential variations in the incidence of dental anomalies based on the gender and nationality of the patients, thereby contributing to a more personalized and effective approach to orthodontic care.

## Materials and methods

This was a retrospective study that used the orthopantomography (OPG) of orthodontic patients. The files of orthodontic patients who completed or underwent treatment in various hospitals of Riyadh Elm University, Riyadh, Saudi Arabia, were included in this study. The patient files were selected based on a convenience sampling methodology. The Research and Innovation Center of Riyadh Elm University, Riyadh, Saudi Arabia, formally approved the study (approval number: FUGRP/2023/306/938/839). Patient files from 2015 to 2023 were retrieved after obtaining permission from the hospital directors of Riyadh Elm University.

Sample size and selection

Three hundred and eighty-four OPG records of the orthodontic patients were calculated based on an effect size of 0.09, an alpha error probability of 0.05, a power (1-β error probability) of 0.95, and a constant proportion of 0.35.

Patients from different nationalities and genders who have received orthodontic treatment at the orthodontic clinics were included in this study based on the following inclusion criteria: healthy male and female patients, aged above 12 years, undergoing or finishing orthodontic treatment, files with complete documentation, and pretreatment OPGs. OPGs of syndromic patients under the age of 12 were excluded from the study.

Evaluation of the OPG

Records include patients' OPGs with dental anomalies, including dilacerated teeth, congenitally missing teeth, supernumerary teeth, hyperdontia, hypodontia, rotation, amelogenesis imperfecta, dentinogenesis imperfecta, and taurodontism. The OPGs were observed, and a single examiner collected data. All the data was collected in an Excel sheet (Microsoft Corporation, Redmond, Washington, United States).

Statistical analysis

The data, initially curated in an Excel spreadsheet, was entered into IBM SPSS Statistics for Windows, Version 25.0 (Released 2017; IBM Corp., Armonk, New York, United States) to facilitate statistical analysis. Descriptive statistical techniques were employed to calculate frequency distributions and percentages for the categorical variables. The relationship between dental anomalies and varying demographic factors such as gender and nationality was explored using the Chi-square test. A p-value threshold of less than 0.05 was established as the criterion for statistical significance across all the tests conducted.

## Results

A total of 384 OPGs of the orthodontic patients were considered for the study, of which 222 (57.8%) belonged to male and 162 (42.2%) to female orthodontic patients. Most patients were Saudi nationals (n=296; 77.1%), compared to non-Saudis (n=88; 22.9%). The study characteristics are shown in Table 1.

**Table 1:** Study characteristics (N=384)

Characteristics		Frequency	Percentage
Gender	Male	222	57.8%
	Female	162	42.2%
Nationality	Saudi	296	77.1%
	Non-Saudi	88	22.9%
Dental anomalies	Dilaceration	23	6.0%
	Congenitally missing	28	7.3%
	Impaction	246	64.1%
	Supernumerary	31	8.1%
	Hyperdontia	29	7.6%
	Hypodontia	0	0.0%
	Rotation	5	1.3%
	Amelogenesis imperfecta	12	3.1%
	Taurodontism	10	2.6%

**Figure 1:**
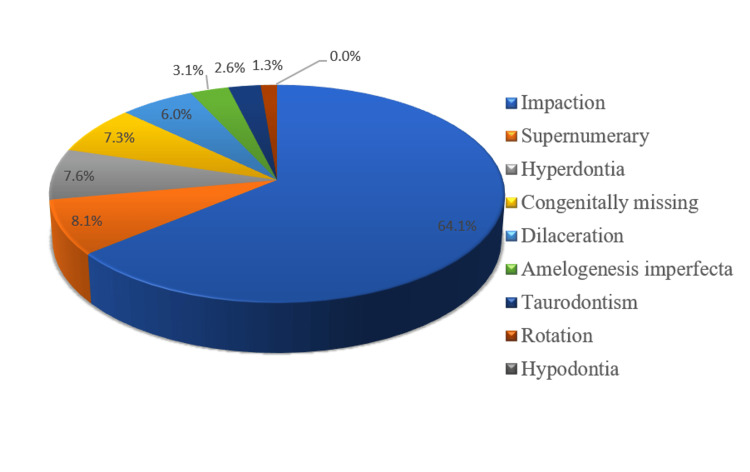
Frequency distribution of the dental anomalies

The prevalence of various dental abnormalities is shown in Figure 1. Impactions were the most common type of dental anomaly observed among orthodontic patients (n=246; 64.1%), followed by the presence of supernumerary teeth (n=31; 8.1%), hyperdontia (n=29; 7.6%), and congenitally missing teeth (n=28; 7.3%). However, the frequency of dilacerated teeth was 23 (6%), amelogenesis imperfecta was 12 (3.1%), taurodontism was 12 (3.1%), and tooth rotations were 5 (1.3%).

Table 2 shows the prevalence of dental anomalies between males and females. The prevalence of different dental anomalies is significantly associated with gender (chi-square = 25.405, p = 0.001). The prevalence of impactions was significantly higher among males (n=154, 69.4%) than females (n=92, 56.8%). Contrarily, a significantly higher prevalence of supernumerary teeth was observed in females (24.8%) than in males (7.3%). Additionally, females showed an insignificantly higher prevalence of dilaceration, hyperdontia, amelogenesis imperfecta, and taurodontism than males. However, congenitally missing teeth and tooth rotations were more commonly found in males than females.

**Table 2:** Comparison of dental anomalies between males and females Note: The superscripts 'a' and 'b' attached to the counts are used to indicate the results of the statistical tests for all dental anomalies. If the counts within a row for a specific anomaly share the same superscript (either 'a' or 'b'), this suggests that there is no statistically significant difference in the proportions of males and females for that anomaly. However, if the counts within a row for an anomaly do not share the same superscript, this indicates that there is a statistically significant difference in the proportions of males and females for that anomaly. A p-value of less than 0.05 indicates a statistically significant difference. Values in the same row and subtable not sharing the same superscript significantly differ at p< 0.05 in the two-sided equality test for column proportions. Cells with no superscript are not included in the test.

Anamolies	Male		Female		Chi-square	p-value
	Frequency	Percentage	Frequency	Percentage		
Dilaceration	11^a^	5.0%	12^a^	7.4%	25.405	0.001
Congenitally missing	21^a^	9.5%	7^a^	4.3%		
Impaction	154^a^	69.4%	92^b^	56.8%		
Supernumerary teeth	7^a^	3.2%	24^b^	14.8%		
Hyperdontia	14^a^	6.3%	15^a^	9.3%		
Hypodontia	0^1^	0.0%	0^1^	0.0%		
Rotation	3^a^	1.4%	2^a^	1.2%		
Amelogenesis imperfecta	5^a^	2.3%	7^a^	4.3%		
Taurodontism	7^a^	3.2%	3^a^	1.9%		

No significant difference in the prevalence of dental anomalies was observed between Saudi and non-Saudi nationals, as shown in Table 3. The comparison of dental anomalies between Saudis and non-Saudis yielded no significant differences (chi-square = 5.657, p = 0.580). However, impaction, supernumerary teeth, hyperdontia, rotation, and amelogenesis imperfecta were higher among Saudi nationals than non-Saudis. In contrast, dilaceration, congenitally missing teeth, and taurodontism were higher among non-Saudis than among Saudis.

**Table 3:** Comparison of dental anomalies between Saudi and Non-Saudi nationals Note: The superscript 'a' attached to the counts is used to indicate the results of the statistical tests for all dental anomalies. If the counts within a row for a specific anomaly share the same superscript, this suggests that there is no statistically significant difference in the proportions of Saudi and Non-Saudi nationals for that anomaly. A p-value of less than 0.05 indicates a statistically significant difference). Values in the same row and subtable not sharing the same superscript are significantly different at p< .05 in the two-sided test of equality for column proportions. Cells with no superscript are not included in the test. Tests assume equal variances.

Anamolies	Saudi		Non-Saudi		Chi-square	p-value
	Frequency	Percentage	Frequency	Percentage		
Dilaceration	14^a^	4.7%	9^a^	10.2%	5.657	0.580
Congenitally missing	21^a^	7.1%	7^a^	8.0%		
Impaction	190^a^	64.2%	56^a^	63.6%		
Supernumerary teeth	25^a^	8.4%	6^a^	6.8%		
Hyperdontia	25^a^	8.4%	4^a^	4.5%		
Hypodontia	0^1^	0.0%	0^1^	0.0%		
Rotation	4^a^	1.4%	1^a^	1.1%		
Amelogenesis imperfecta	10^a^	3.4%	2^a^	2.3%		
Taurodontism	7^a^	2.4%	3^a^	3.4%		

Table 4 shows the different types of impactions that were observed across the assessed sample size.

**Table 4:** Types of impactions that were observed in the assessed sample size

Type of Tooth	Type of Impaction	Number of Impactions	Percentage of Total Impactions
Wisdom Teeth (Third Molars)	Complete Mesial	89	36.6%
	Partial Mesial	57	24.4%
	Vertical	34	12.2%
Canines	Complete Mesial	16	8.1%
	Partial Mesial	14	4.1%
	Distal	7	4.1%
Premolars	Complete Mesial	13	4.1%
	Partial Mesial	5	2.0%
	Distal	5	2.0%
Incisors	Complete Mesial	3	1.2%
	Partial Mesial	2	0.8%
	Distal	1	0.4%

## Discussion

The significance of our investigation's findings lies predominantly in their potential to inform more accurate diagnoses and treatment plans in orthodontic care. Recognizing patterns of dental anomalies related to gender and nationality could enable clinicians to predict patients' risks for certain conditions and tailor their care accordingly. These findings also underline the importance of further research to understand the genetic, hormonal, and environmental factors that may contribute to these disparities. Such knowledge could open up new avenues for preventive and personalized medicine in dentistry and improve outcomes for patients.

The findings from the current study indicate a significant gender-based discrepancy in the occurrence of various dental anomalies. The study enlisted a higher proportion of male participants, which is unusual for orthodontic studies as females typically seek more orthodontic care due to a higher concern for aesthetics [10,11]. The study found dental conditions like dilaceration and supernumerary teeth to be more common in females, while males were more likely to exhibit congenital absence and impactions of teeth. This is consistent with previous research, such as the study conducted by Afifty and Zawawi [1], reinforcing the validity of these findings. Additionally, taurodontism, a condition characterized by a change in tooth shape, was found to be more prevalent in males, aligning with the findings of a study by Rakhshan [12]. Notably, these gender-based discrepancies could have significant implications for the field of orthodontics. Recognizing these patterns can inform more accurate diagnoses and treatment plans. For instance, if a female patient presents with a dental anomaly, the likelihood of it being dilaceration or supernumerary teeth might be higher compared to other conditions. As for the potential causes of these gender-specific disparities, one possibility suggested by the study is genetic factors, although further research would be needed to confirm this hypothesis. If confirmed, it could open up new avenues for genetic testing or personalized medicine in dentistry, enabling clinicians to predict a patient's risk for certain dental anomalies based on their sex. However, the skewed gender ratio in the study sample could have introduced some bias into the results. The researchers mitigated this by conducting separate evaluations for each gender, but the imbalance remains a limitation of the study. Future studies should aim for a more balanced gender distribution to ensure the results are generalizable across the population. The study's findings also align with a similar study conducted in Egypt [11], suggesting that these patterns might be consistent across different populations and cultural contexts. This could underscore the potential universal applicability of these findings, although more research is needed in various demographic and geographic settings to confirm this.

In the present study, we found patterns that are consistent with research by Guttal et al. [13], which highlighted a higher occurrence of taurodontism in males. This pattern suggests that hormonal fluctuations may play a role in the manifestation of this dental anomaly. However, our investigation did not find any significant gender-related differences in the incidence of hypodontia or amelogenesis imperfecta. Recognizing such disparities is vital for the accurate diagnosis and management of dental abnormalities, especially in patients who exhibit a gender-associated predisposition to a specific anomaly. Nevertheless, our findings underline the pressing need for further research to shed light on the genetic, hormonal, and environmental factors that may underpin these gender-related differences.

When the findings of the current study were compared with existing research on dental anomaly prevalence across different populations, some discrepancies emerged. For example, a significantly higher prevalence of congenital tooth absence was noted in the non-Saudi group compared to the Saudi group. This finding contradicts previous studies by Younes et al. [14] and Garot et al. [15], which reported a higher incidence of congenitally missing teeth in Middle Eastern populations, potentially due to genetic, environmental, and cultural factors influencing dental maturation. On the other hand, our data revealed a higher incidence of hyperdontia in the Saudi population, which is in line with a prior study by Al-Sebaei et al. [16]. This similarity suggests that a genetic predisposition towards hyperdontia could result from changes in the expression of certain genes involved in tooth development. Our study also found a higher prevalence of amelogenesis imperfecta in the Saudi group compared to the non-Saudi group, which is consistent with previous research conducted in Middle Eastern populations [16].

Nonetheless, it is crucial to acknowledge that the prevalence of dental anomalies can exhibit significant variation within a population, influenced by factors such as age, gender, and environmental conditions [17]. This consideration warrants caution in extrapolating the findings of this study to the broader Saudi population and populations beyond Saudi Arabia. Further investigations encompassing larger and more diverse sample sizes are necessitated to corroborate these findings and deepen our understanding of dental anomalies across different populations. Therefore, further studies should make use of the power of three-dimensional (3D) imaging in orthodontics. In other words, cone-beam computed tomography (CBCT) images should be our diagnostic tool in future studies about dental anomalies.

While our study offers meaningful data about the incidence of dental irregularities in orthodontic patients, it is not exempt from constraints. First, our analysis was based on a fairly limited cohort of OPGs from orthodontic patients. Such a sample size might not be sufficiently large to accurately reflect the wider population, thereby potentially curtailing the reach of our findings. Second, our research relied solely on one kind of imaging technique, namely OPGs. This method might not reveal every dental anomaly, particularly those that might be more effectively displayed using other imaging protocols. Moreover, an important point to consider is the non-utilization of available CBCT images that are readily accessible in most orthodontic departments. CBCT provides a more comprehensive view of dental structures and could potentially identify a wider range of dental anomalies. Future research should incorporate CBCT images to enhance the detection and understanding of dental anomalies, thereby providing a more robust and detailed analysis. Fourth, our study did uncover significant correlations between gender and the incidence of particular dental anomalies, such as tooth impaction and supernumerary teeth. However, we did not uncover substantial disparities in the incidence of dental anomalies between Saudi and non-Saudi nationals. This calls into question the prevailing supposition that nationality or ethnic background might have an influence on the manifestation of certain dental anomalies. This area of research could benefit from further investigation.

## Conclusions

This study's findings suggest differences in specific dental anomalies between genders and Saudi and non-Saudi nationals. These findings highlight the significance of population-specific variables in identifying and managing dental abnormalities. A noteworthy discovery with important clinical ramifications is the high prevalence of impacted teeth, congenitally missing teeth, and a lack of hypodontia in the Saudi population. Early identification and treatment are crucial for improving outcomes because these dental anomalies can significantly damage the oral health and quality of life of those affected.

## References

[1] Afify AR, Zawawi KH (2012). The prevalence of dental anomalies in the western region of Saudi Arabia. ISRN Dent.

[2] Lee JH, Kinabalu K (2020). Prevalence of dental anomalies among orthodontic patients in South-East Sabah. Dent J Malays.

[3] Syriac G, Joseph E, Rupesh S, Philip J, Cherian SA, Mathew J (2017). Prevalence, characteristics, and complications of supernumerary teeth in nonsyndromic pediatric population of south India: a clinical and radiographic study. J Pharm Bioallied Sci.

[4] Gill DS, Barker CS (2015). The multidisciplinary management of hypodontia: a team approach. Br Dent J.

[5] Ericson S, Kurol J (1986). Radiographic assessment of maxillary canine eruption in children with clinical signs of eruption disturbance. Eur J Orthod.

[6] Patil S, Maheshwari S (2014). Prevalence of impacted and supernumerary teeth in the North Indian population. J Clin Exp Dent.

[7] Khalaf K, Miskelly J, Voge E, Macfarlane TV (2014). Prevalence of hypodontia and associated factors: a systematic review and meta-analysis. J Orthod.

[8] ALumaid J, Buholayka M, Thapasum A, Alhareky M, Abdelsalam M, Bughsan A (2021). Investigating prevalence of dental anomalies in eastern province of Saudi Arabia through digital orthopantomogram. Saudi J Biol Sci.

[9] Yassin SM (2016). Prevalence and distribution of selected dental anomalies among Saudi children in Abha, Saudi Arabia. J Clin Exp Dent.

[10] Lagorsse A, Gebeile-Chauty S (2018). Does gender make a difference in orthodontics? A literature review [Article in French]. Orthod Fr.

[11] Al-Jabaa AH, Aldrees AM (2013). Prevalence of dental anomalies in Saudi orthodontic patients. J Contemp Dent Pract.

[12] Rakhshan V (2015). Congenitally missing teeth (hypodontia): a review of the literature concerning the etiology, prevalence, risk factors, patterns and treatment. Dent Res J (Isfahan).

[13] Guttal KS, Naikmasur VG, Bhargava P, Bathi RJ (2010). Frequency of developmental dental anomalies in the Indian population. Eur J Dent.

[14] Younes SA, al-Shammery AR, el-Angbawi MF (1990). Three-rooted permanent mandibular first molars of Asian and black groups in the Middle East. Oral Surg Oral Med Oral Pathol.

[15] Garot E, Rouas P, Somani C, Taylor GD, Wong F, Lygidakis NA (2022). An update of the aetiological factors involved in molar incisor hypomineralisation (MIH): a systematic review and meta-analysis. Eur Arch Paediatr Dent.

[16] Al-Sebaei M, Sindi MA (2023). A knowledge and practice survey among dentists in saudi arabia analysing myths and misconceptions in dentistry and oral surgery: what do dentists believe?. Cureus.

[17] Souza-Silva BN, Vieira WA, Bernardino ÍM, Batista MJ, Bittencourt MA, Paranhos LR (2018). Non-syndromic tooth agenesis patterns and their association with other dental anomalies: a retrospective study. Arch Oral Biol.

